# Nanomaterials-based vaccines to target intracellular bacterial pathogens

**DOI:** 10.3389/fmicb.2022.1040105

**Published:** 2022-11-18

**Authors:** Megan A. Files, Kadin M. Kristjansson, Jai S. Rudra, Janice J. Endsley

**Affiliations:** ^1^Department of Microbiology and Immunology, Galveston, TX, United States; ^2^Institute of Translational Science, University of Texas Medical Branch, Galveston, TX, United States; ^3^Department of Medicine, School of Medicine, Seattle, WA, United States; ^4^Department of Chemistry, Smith College, Northampton MA, United States; ^5^Department of Biomedical Engineering, McKelvey School of Engineering, Washington University in St. Louis, St. Louis, MO, United States

**Keywords:** vaccines, nanomaterials, peptide nanofibers, intracellular bacterial pathogens, *Mycobacterium tuberculosis*

## Abstract

Development of novel immunization approaches to combat a growing list of emerging and ancient infectious agents is a global health priority. Intensive efforts over the last several decades have identified alternative approaches to improve upon traditional vaccines that are based on live, attenuated agents, or formulations of inactivated agents with adjuvants. Rapid advances in RNA-based and other delivery systems for immunization have recently revolutionized the potential to protect populations from viral pathogens, such as SARS-CoV-2. Similar efforts to combat bacterial pathogens, especially species with an intracellular niche, have lagged significantly. In the past decade, advances in nanotechnology have yielded a variety of new antigen/adjuvant carrier systems for use in vaccine development against infectious viruses and bacteria. The tunable properties of nanomaterial-based vaccines allow for balancing immunogenicity and safety which is a key hurdle in traditional antigen and adjuvant formulations. In this review, we discuss several novel nanoparticle-based vaccine platforms that show promise for use against intracellular bacteria as demonstrated by the feasibility of construction, enhanced antigen presentation, induction of cell mediated and humoral immune responses, and improved survival outcomes in *in vivo* models.

## Introduction

Vaccination is one of the most successful public health initiatives and the global response to the recent SARS-CoV2 pandemic highlights the advances in antiviral countermeasures. However, vaccines targeting intracellular bacterial pathogens of clinical importance or biowarfare concern, such as *Mycobacterium* spp., *Burkholderia spp.*, *Listeria monocytogenes (L. monocytogenes)*, *Chlamydia trachomatis (C. trachomatis)*, *Borrelia burgdorferi (B. burgdorferi)*, *Brucella abortus (B. abortus)*, and *Shigella flexneri* (*S. flexneri*) among others are significantly underdeveloped. Persistence of disease caused by these intracellular pathogens, such as tuberculosis, melioidosis, listeriosis, chlamydia, brucellosis, and shigellosis, are due to endemic status of infections in many regions of the world. Clinical manifestations of these diseases range widely and affect multiple systems, including respiratory, gastrointestinal, urogenital, reproductive, and dermal.

The success of intracellular bacterial pathogens lies in their ability to infect and survive inside host cells and arrest the development of protective immunity. Often, host targets are antigen-presenting cells (APC) or epithelial stromal cells where the bacteria take advantage of the host cell nutrients, environment, potential for locomotion, or other characteristics. While residing inside host cells, the bacteria can avoid detection through multiple mechanisms including antigenic variation, secretion of immunomodulators, or evasion of killing mechanisms through suppression of host immune responses (Palmer et al., 2016; Chai et al., 2020; Leseigneur et al., 2020; Syed and Wooten, 2021). Occupation of the host cell thus provides a protected niche that permits some bacteria to establish persistent or chronic infections that are difficult to treat (Thakur et al., 2019). Although these bacterial infections are often treatable with appropriate use of antibiotics, several drug-resistant strains complicate treatment and contribute to continued transmission while new classes of antibiotics have been slow to reach the clinic.

Traditional vaccination approaches utilize attenuated or inactivated organisms and subunit vaccines. Live attenuated vaccines, e.g., *Mycobacterium bovis* bacille Calmette- Guerin (BCG) and the oral typhoid vaccine, can be risky in vulnerable populations (e.g., immunocompromised individuals), or otherwise have inadequate safety profiles (Acosta et al., 2005; Hanley, 2011). Inactivated organisms are generally low risk, although the inactivation process sometimes results in loss of immunogenicity. Subunit vaccines are much safer than live attenuated vaccines but lack substantial immunogenicity and require co-administration with adjuvants. Delivering robust cell-mediated and humoral responses while maintaining safety is a challenge in vaccine design. This has prompted the development of novel nanoparticle (NP) vaccine platforms as delivery vehicles for antigen and other immunomodulators against numerous intracellular pathogens.

Targeting professional APC of the innate immune system is an important means to elicit optimum responses to vaccination. APC such as dendritic cells (DC) and macrophages encounter and take up foreign antigen during the early stages of infection. Typically, antigenic peptides are processed through lysosomal degradation and presented on the surface of cells in the context of MHC molecules (Kotsias et al., 2019). Activation of co-stimulatory molecules and cytokines further optimize the interactions of APC with T cells of the acquired immune system. Antigen presentation can be subverted by several intracellular pathogens during natural infection, including *Mycobacteria spp* (Griffiths et al., 2016), *Burkholderia spp* (Syed and Wooten, 2021), and *L. monocytogenes* (Corr and O’Neill, 2009), through a diverse array of mechanisms, which then limit cell-mediated and humoral immune responses. Some myeloid-derived innate immune cells, particularly monocytes and macrophages, have demonstrated a form of memory called trained innate immunity (TII; Geckin et al., 2022). Following stimulation with potent immune activators, such as β-glucan, lipopolysaccharide (LPS), oxidized-low density lipoprotein (ox-LDL), and BCG, the cell undergoes epigenetic modification that results in subsequent functional changes. Upon restimulation with unrelated antigens, trained monocytes and macrophages exhibit an enhanced inflammatory state (Steigler et al., 2019; Geckin et al., 2022).

Cell-mediated immunity (CMI) predominates in the protective response against intracellular bacteria, while the role of humoral immunity is less understood. Vaccine efforts to prevent disease due to intracellular bacterial pathogens are thus primarily focused on generation of memory T cells. Naïve T cells need to be primed by APC to generate protective responses, but memory T cells, which reside in circulation, in tissues, and in lymphoid organs, can rapidly respond to pathogen insult (Kaech et al., 2002). Following activation by specific antigen, T cells produce effector cytokines that orchestrate actions of other immune cells and can mediate direct cytotoxic activity against infected cells. In general, CD8^+^ T cells are specialized for cytotoxic activity while CD4^+^ T cells have superior helper function through cytokine production. IFN-γ is an especially significant effector cytokine produced by memory T cells that coordinates interactions between innate and adaptive immune populations. Importantly, IFN-γ drives inflammatory and nitrositive stress responses needed to control intracellular bacterial infections (Flynn et al., 1993; Dellacasagrande et al., 2002; Rottenberg et al., 2002; Jehl et al., 2012; Assani et al., 2014).

Several other cytokines have also been shown to promote critical, yet varied T cell responses associated with protective outcomes. For example, immunity to *Mycobacterium tuberculosis* (Mtb) infection is associated with activity of T helper cells that predominantly produce IFN-γ (Th1) or IL-17 (Th17) (Flynn et al., 1993; Ogongo et al., 2021); thus, these T cell populations are a primary target of investigational TB vaccines (Kaufmann et al., 2017; Nieuwenhuizen and Kaufmann, 2018). A polyfunctional cytokine profile, especially those including IFN-γ, IL-17A, and TNF-α, as well as the tissue resident status of T cell memory pools, is increasingly understood to correlate with protective immune memory (Sakai et al., 2014; Khakhum et al., 2021; Files et al., 2022). The complexity of the cellular immune response, coupled with the difficulty of identifying protective antigens that elicit protective responses, presents a challenge for rational vaccine design against intracellular pathogens (Titball, 2008). Measurements of T cell responses as immune correlates of protection are a difficult standard to define compared to antibody titers (Casadevall and Fang, 2020).

Humoral immunity, as determined by generation of protective antibodies, is an important and routine endpoint for defining the efficacy of vaccines for extracellular bacterial and many viral pathogens. Vaccine-induced humoral immunity against extracellular bacteria often depends on antibody-mediated targeting of specific antigens that block infectivity or other mechanisms of pathogenesis. An important example is the prevention of diphtheria and cholera by vaccines that elicit antibodies that neutralize toxin activity (Ghattas et al., 2021). Antibodies also have functional properties related to the capacity to opsonize bacteria, activate the complement cascade, and modulate effector functions of immune cells. Humoral immunity contributes a minor, though emerging, role in the acquired immune response to intracellular bacteria (Casadevall and Fang, 2020). An example includes anti-listeriolysin O antibodies which gain entry to the cell as a mechanism to prevent *L. monocytogenes* infection (Edelson and Unanue, 2001). Other literature further suggests that development of Mtb-specific antibodies may improve tuberculosis outcomes after infection and can be a mechanism for protection generated through BCG vaccination (Achkar et al., 2015; Bitencourt et al., 2022). The immune basis for this protection lies in the development of functional bias that enhances antibody binding to Fc receptors (FcR) and promotes antimicrobial activity of innate immune cells such as macrophages (Lu et al., 2016; Bitencourt et al., 2022). Antibody engagement of the FcR can activate host cell defense by directing pathogens to lysosomal degradation (Joller et al., 2010). Several vaccines candidates discussed in this review have been shown to elicit antibodies, and in some cases have been shown to contribute to protective efficacy through poorly understood mechanisms (Kamp et al., 2020; Khakhum et al., 2021). The protective effects of antibodies against intracellular bacteria are thus an important area of research in the context of novel vaccine platforms (Joller et al., 2010; Casadevall, 2018).

## Emerging vaccine approaches

**Figure 1:**
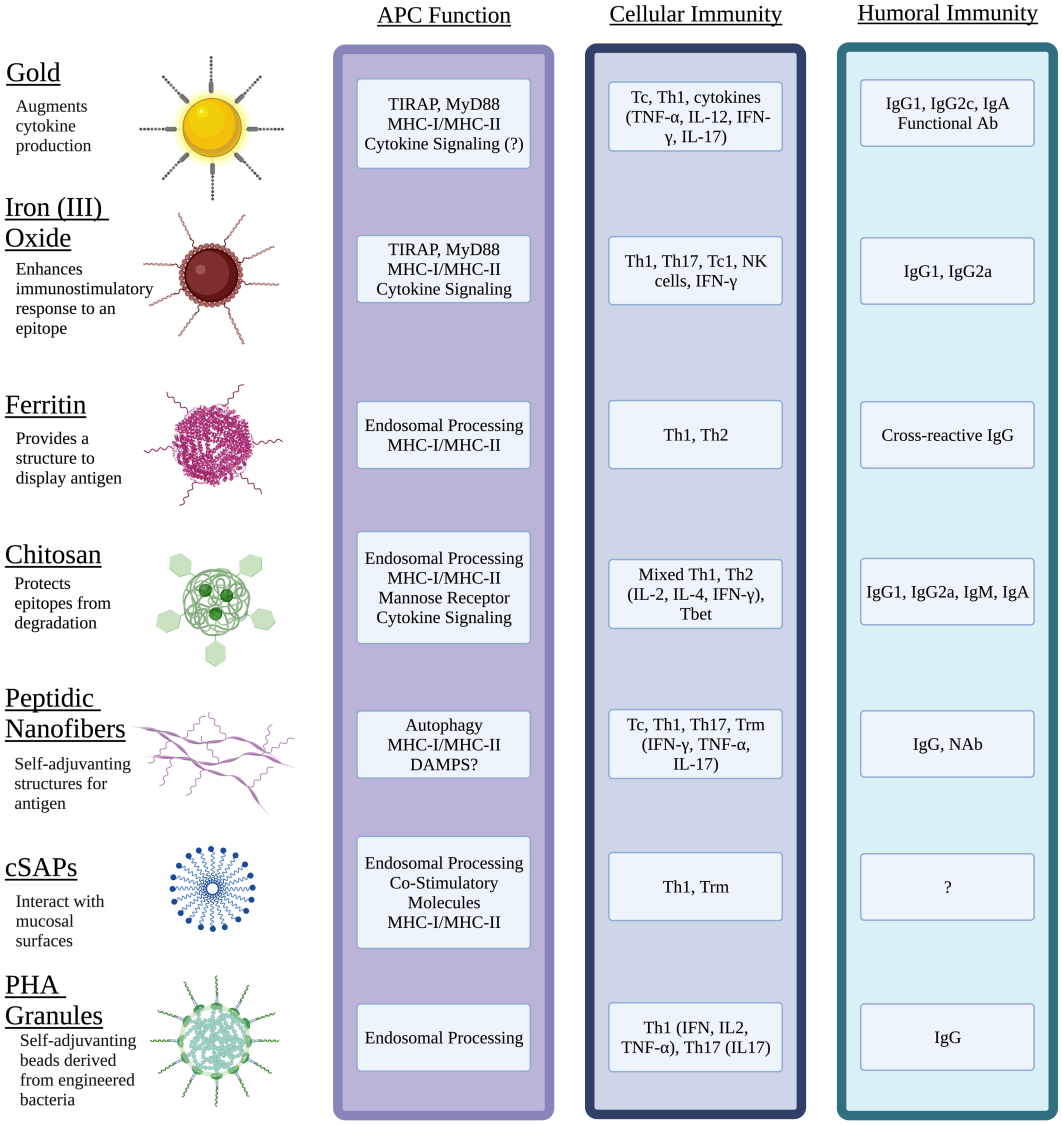
Nanomaterial vaccines target antigen presenting cells (APC) and generate immune memory to intracellular bacterial pathogens. Physical properties of different NPs optimize epitope display, protect antigen integrity, facilitate interactions with mucosal surfaces, and target innate immune pathways of APC. Unique features of NPs can be exploited to activate cytokine signaling through pathogen pattern receptors (e.g., TLR, CLR) and direct antigen to exogenous and/or endogenous processing pathways that present epitopes *via* MHC I and MHC II. The resulting interactions between mature APC and naïve lymphocytes of the acquired immune system can generate potent humoral immunity including mucosal antibody (Ab) such as IgA, neutralizing Ab (NAb) and functional Ab that promote antimicrobial activity of innate immune cells. Diverse populations of antigen-specific T cells with tissue resident status (Trm), cytotoxic T cell (Tc) activity, and the potential to produce effector cytokines (IFN-γ, IL-17) with roles in clearance of intracellular pathogens can be generated through choice of NP, antigen(s), and adjuvant. Image created with Biorender.com.

In recent years, novel vaccine platforms that address the challenges associated with traditional vaccines have been developed. These techniques include incorporating liposomes, virus-like particles, micelles, DNA, mRNA, and nano-scale particles, cages, or fibers. Nanoparticles can be derived from biological materials or can be inorganic composites or synthetic, such as iron oxide or gold NPs. Additionally, NPs have been shown to be effective antigen carriers that improve uptake by APC and are easily customizable in terms of valency, size, degradation, targeting, and other features which can alter the pharmacokinetics. The use of liposomes, micelles, and emulsions has been extensively covered elsewhere, thus this review will focus primarily on prophylactic vaccine designs that incorporate nanostructures of gold, iron oxide, ferritin, charge switching adjuvants, polyesters, chitosan, and designed peptides (Figure 1).

### Gold nanoparticles

The shape and size of gold (Au) NP (Au-NP) can be easily customizable and are reliably functionalized to carry multiple proteins or DNA antigens and adjuvants. Au-NP have been used in several vaccines due to their relative inertness, which minimizes toxicity. However, the route of administration and shape of AuNPs impacts the expression of some cytokines (Mateu Ferrando et al., 2020) as well as biodistribution which can be important for targeting intracellular pathogens. This has been demonstrated in a model of melioidosis, an important disease with a high fatality rate caused by infection with *Burkholderia pseudomallei* (Bpm). Bpm is acquired through several routes and has complex interactions with the human host that complicate vaccine design. Interestingly, a unique design comprised of Bpm OpcP, OpcP1, and LPS utilizing the AuNP platform has shown strong promise as a vaccine (Tapia et al., 2021). OpcP and OpcP1 are predicted to form porins in the outer membrane and were identified as vaccine antigen candidates through a reverse vaccinology bioinformatic analysis (Tapia et al., 2021). In a murine model of Bpm, mucosal immunization led to protection in 90% of animals and significantly reduced bacterial burden and dissemination following challenge (Tapia et al., 2021). Protection was associated with development of antigen-specific lung IgA, serum IgG, and polyfunctional cytokine responses from splenocytes, including IFN-γ, TNF-α, IL-2, IL-17, and IL-10. Sera containing antibodies from vaccinated animals elicited by the AuNP vaccine had opsonizing activity and further promoted opsonophagocytosis by macrophages *in vitro*. Isotyping revealed predominantly IgG2c and IgG1, known to be associated with mixed Th1/Th2 responses and protection against Bpm (Tapia et al., 2021). The same research group previously showed that a different AuNP vaccine (conjugated to different protein antigens) induced 50% protection in non-human primates (Gregory et al., 2015). These are important immunization outcomes, as human convalescent patients have high LPS-specific antibody levels as well as increased numbers of CD4^+^ and CD8^+^ T cells (Sengyee et al., 2019).

*L. monocytogenes* is the causative agent of listeriosis, which most often occurs from ingestion of contaminated food. Besides the unpleasant symptoms of food poisoning, listeriosis can cause miscarriage during early pregnancy (Lamont et al., 2011). In a model of listeriosis, Au NPs conjugated to the CD8^+^ T cell epitope from listeriolysin O (LLO_91-99_), which is an important mediator of immune evasion and replication of *L. monocytogenes,* were shown to improve DC-mediated antigen presentation to T cells when compared with DCs loaded with antigen alone. Intravenous (i.v.) vaccinations with DCs loaded with GNP-LLO_91-99_ conferred ~94% protection from challenge with *L. monocytogenes* measured by the reduction in bacterial burden. Splenic IFN-γ^+^ CD8^+^ T cells and DCs were also observed to increase in vaccinated animals, suggesting enhanced trafficking and antigen presentation in lymphoid organs (Rodriguez-Del Rio et al., 2015). Animals immunized with DC-GNP-LLO_91-99_ had increased levels of cytokines associated with a Th1 bias including MCP-1, TNF-α, IFN-γ, and IL-12; however, it was IL-12 that was uniquely correlated with the enhanced protection conferred by the vaccine. The authors also sought to replicate the effect of DCs on T cell function by using unconjugated Advax, a polysaccharide adjuvant, in place of DCs. Interestingly, an intraperitoneal (i.p.) vaccination with GNP-LLO_91-99_ plus Advax yielded similar results as the DC vaccine, including an increased frequency of mature DCs, antigen-specific, and total IFN-γ^+^ CD8^+^ T cells in the spleen. Overall, formulating the GNP-LLO_91-99_ vaccine with Advax protected animals from virulent challenge associated with development of strong Th1 responses (Rodriguez-Del Rio et al., 2015).

In a follow up study, a second Au-NP vaccine conjugated to *L. monocytogenes* GADPH_1-22_ was formulated and the two vaccines were then evaluated for their efficacy to prevent listeriosis during pregnancy (Calderón-Gonzalez et al., 2016). Pups born to non-vaccinated, and *L. monocytogenes*-infected mice were fewer in number and displayed significant weight loss, reduced movement, and had lighter and more wrinkled skin as compared to the non-infected controls. These results suggested significant changes in nervous system and cutaneous development, which was further supported by high bacterial burden in liver and microglial cells in brain of pups from non-vaccinated mice. There were no viable colony forming units (CFU) of bacteria detected in liver and spleen of mothers vaccinated with either GNP-LLO_91-99_ or GNP-GAPDH_1-22_ vaccines. Similar to human cases of neonatal meningitis, increased levels of IL-6 and TNF-α in microglial cells were observed in non-vaccinated animals. Vaccination with GNP vaccines induced an increase in IL-12 and a reduction in IL-6 in response to challenge. These findings demonstrate the safety of these GNP vaccines as evidenced by healthy pups born to vaccinated mothers. Lack of detectable bacteria in the brain or liver of pups born to vaccinated and challenged mice further demonstrated protective efficacy of the GNP vaccine (Calderón-Gonzalez et al., 2016). In more recent investigations, vaccination with adjuvanted (DIO-1 or Advax) GNP-GADPH_1-22_ was further shown to prevent listeria-induced effects on microglia including reduction of apoptosis, a shift toward a Th1 response, and an increased TNF-α/IL-6 ratio. These immune outcomes were observed concurrently with reduced bacterial dissemination when compared to non-vaccinated mice (Calderon-Gonzalez et al., 2017).

### Iron (III) oxide nanoparticles

Iron oxide NPs have been used extensively for biomedical applications ranging from magnetic imaging to drug and vaccine delivery. They are not only an effective vehicle for vaccine delivery, but also possess immunomodulatory properties. Pusic, et al. demonstrated efficient uptake of iron oxide NPs by murine DCs and macrophages, followed by an increased expression of costimulatory molecules, such CD86, and numerous chemokines and cytokines including IFN-γ, TNF-α, IL-1β, and IL-6 (Pusic et al., 2013). Further evidence that iron NP-based vaccine constructs activate cytokines and costimulatory molecules associated with recruitment of immune cells and priming of T cells was also demonstrated by Neto et al. (2018). A comprehensive understanding of the outcomes of interactions of iron oxide NPs with immune cells is lacking to date and represents an important area for further research (Yu et al., 2012; Feng et al., 2018).

Iron oxide NPs can be modified by the incorporation of other metal ions, such as manganese. Analogous to iron oxide, manganese induces proinflammatory responses through NF-kB, including IL-6 and TNF-α production, as well as superoxide activity (Filipov et al., 2005). An example of this approach is use of MnFe_2_O_4_ NPs in a subunit vaccine with a commonly used fusion protein composed of immunodominant Mtb peptide antigens (Ag85c, MPT51, and HspX) (de Sousa et al., 2012). Importantly, the Ag85c is a major secreted antigen in active TB, and immune cells from individuals with either active or latent TB respond to MPT51 and HspX (de Sousa et al., 2012). In a series of studies, immunogenicity and protection in mice vaccinated with this construct were observed to vary with route and schedule of immunization (Neto et al., 2018). A subcutaneous (s.c.) vaccination induced a predominant Th1 response in both the lung and spleen as well as a strong humoral response that included significant and balanced production of IgG1 and IgG2a. Vaccination through the intranasal (i.n.) route induced antigen-specific Th1, cytotoxic T cells that produce Th1 cytokines (Tc1), and Th17 responses, primarily in the lung. This approach, however, failed to induce the IgG1 and IgG2a antibodies that were observed following the s.c. dosing regimen. When a mixed vaccination strategy was employed (two s.c. doses, followed by one i.n. vaccination), there was a pronounced Th1 response in both lung and spleen. However, the enhanced Th17 responses in the lung generated by i.n. vaccination was not observed.

Differences in disease endpoints following challenge further support the efficacy of MnFe_2_O_4_ NP vaccines. Following an Mtb challenge, the s.c. vaccination regimen reduced the bacterial load of Mtb in the lungs compared to non-vaccinates, but not compared to a group vaccinated s.c. with BCG. In a histological evaluation of the lungs, the mice given s.c. vaccinations exhibited fewer lung lesions compared with unvaccinated controls but displayed some areas with mononuclear infiltration. Interestingly, larger areas of mononuclear cell infiltration were observed post-challenge in lung of mice vaccinated through the i.n. route. The mixed vaccination regimen induced less protection against Mtb challenge compared to both BCG and the s.c. vaccination (Neto et al., 2018). Overall, this study demonstrated that iron oxide NP vaccines can induce cell-mediated and humoral immune responses, which may be protective as demonstrated by the reduced bacterial burden in the lungs of vaccinated mice. Importantly, this study also shows that immune responses generated by iron oxide NP-based constructs can be influenced by the route of vaccination. The development and functionality of vaccine-induced antibodies was not evaluated in this study and requires further exploration.

Yu, et al. further demonstrated the capacity of iron oxide NPs to induce protective immune responses to Mtb. A construct of Fe_3_O_4_ NPs coated with glutamic acid (Fe_3_O_4_-Glu NP) were conjugated to a DNA vector vaccine through polyethyleneimine (PEI). The DNA vaccine vector contains sequences for a fusion protein of Ag85A and ESAT6, and IL-21 on a pIRES backbone (Yu et al., 2012). Ag85A and ESAT6 are both protective T cell antigens that are widely used in tuberculosis vaccine design. The NP acts as the carrier, and IL-21 acts as an adjuvant by stimulating the production of IFN-γ and enhancing antigen presentation of the fusion protein by macrophages and DCs to lymphocytes. IL-21 is also involved in natural killer (NK) cell activation and is important in B cell differentiation and antibody class switching (Spolski and Leonard, 2014). Mice vaccinated with the NP vaccines carried a significantly lower bacterial burden (~1 log) in their lungs compared with BCG vaccinees after 8 weeks of infection. Similar to BCG, vaccination with the non-conjugated DNA vaccine also improved bacterial burden in lung compared with negative control groups that received mock vaccination with conjugated and unconjugated plasmid or PBS. Vaccination with the NP conjugated DNA vaccines also resulted in significantly lower pathology scores, which considered peribronchiolitis, perivasculitis, alveolitis, and presence of granulomas, compared to BCG and all other groups (Yu et al., 2012). Additionally, animals immunized with the NP DNA vaccines demonstrated an increased cytotoxic activity of NK cells, splenocytes, and CD8^+^ splenocytes compared with all other groups.

To date, the specificity of these responses to the Mtb DNA Fe_3_O_4_-Glu NP vaccine has not been described and is important to understanding the basis for the protective outcomes. ESAT6- and Ag85A-specific antibodies in serum were measured for each immunized group at 2 weeks after the final immunization and demonstrated enhanced antibody production in both groups that received DNA vaccine. Antibody levels were greatest in animals immunized with conjugated NP compared to those that received BCG or the unconjugated DNA vaccine (Yu et al., 2012). Together these data suggest that the Fe3O4-Glu NPs effectively enhanced the immunostimulatory effects of a DNA vaccine, and improved outcomes in comparison with BCG. An important consideration for use of iron NP for vaccination is the potential to cross the blood brain barrier. Access to the CNS is an important feature of iron NP that facilitates magnetic-based neural imaging while also allowing uptake by tissue APC. Interestingly, use of iron oxide NP as part of a CNS imaging approach was shown to suppress IL-1β produced by microglial cells in response to LPS exposure (Wu et al., 2013). Stimulation with LPS typically increases IL-1β secretion through activation of pro-IL-1β expression and the inflammasome (Martin-Sanchez et al., 2016). The suppressive effect of iron oxide NPs on microglia-derived IL-1β was shown to be mediated through inhibition of the secretory lysosomal pathways of cytokine processing (Wu et al., 2013). Vaccine constructs based on iron NP thus need to consider access to, and innate immune responses of, APC in different tissue compartments.

### Ferritin nanostructures

Ferritin is a naturally derived product and can be found in nearly all living organisms. In its natural role, the primary function of ferritin is to store up to 4,500 iron atoms in a soluble, non-toxic form. Similar to virus-like particles (VLPs), ferritin is comparable in size and shape relative to pathogens that mammalian cells normally encounter. Due to its many desirable physical properties, such as thermal and pH stability, uniformity, biodegradability, and biocompatibility, ferritin is an attractive platform for drug delivery and vaccine design. It is additionally amenable to functionalization through genetic or chemical surface conjugation and is relatively low-cost to produce at a large scale (Rodrigues et al., 2021).

The use of ferritin nanocages to display antigenic epitopes has been demonstrated utilizing *Heliobacter pylori* ferritin (Hpf), a common ferritin scaffold, which displays high copy numbers of antigen epitopes to be presented to APC. To determine the innate immune stimulating potential of ferritin nanocages, Han, et al. evaluated ferritin NPs bearing OT-I (chicken egg ovalbumin 257–264) or OT-II (chicken egg ovalbumin 323–339) antigens and their interactions with DCs (Han et al., 2014). The DCs were shown to efficiently phagocytose ferritin protein cages (FPCN), then the NPs were trafficked and processed in endosomes, and antigen was presented on the surface. Following antigen presentation, FPCN-loaded DCs presented antigen to T cells as evidenced by the proliferative response of OT-I and OT-II specific T cells both *in vitro* and *in vivo*. These T cells then differentiated into functional Th1 and Th2 cells with distinct cytokine expression profiles and cytolytic activity (Han et al., 2014). These results demonstrated that antigen-loaded ferritin NPs can be effectively used to target professional APC and elicit antigen-specific T cell responses.

A recent study highlighted the utility of FPCN to elicit antibodies against known protective epitopes. The immunodominant outer surface protein A (OspA) of *B. burgdorferi* B31 was genetically modified to improve its functionality and fused to the N-terminus of ferritin derived from *Heliobacter pylori* (Hpf; Kamp et al., 2020). Monovalent Hpf-OspA constructs adjuvanted with Ribi, AddaVax, or AF03, generated high titers of OspA-specific antibodies. A four-fold increase in titers was observed in animals given the Hpf-OspA vaccine compared to those immunized with Recombitek, the canine Lyme disease vaccine used as a positive control. A hexavalent vaccine consisting of OspA fusion proteins from six serotypes was generated by fusing proteins to the N-terminus of Hpf and combining each construct in equimolar amounts. Controls utilized in this experiment consisted of monovalent Hpf-OspA of one serotype mixed with 5 equimolar amounts of control ferritin particles. This vaccine elicited high antibody titers active against all six serotypes, demonstrating broad cross-reactivity. In a challenge model, all mice that received the hexavalent OspA vaccine were protected from infection after exposure to *B. burgdorferi*-infected ticks, while those that received the ferritin particle negative control were not protected.

To more closely model human immune responses, the hexavalent OspA vaccine was administered to non-human primates (NHP) in combination with the AF03 adjuvant. The adjuvanted hexavalent vaccine induced robust and sustained antibody responses 200-fold of that induced by Recombitek. To further enhance the immune response, a TLR7/8 agonist, 3 M-012, was directly conjugated to the ferritin in the hexavalent vaccine. These NP were estimated to display up to 24 molecules of agonist and demonstrate the highly customizable feature of ferritin. Animals vaccinated with the 3 M-012 conjugated hexavalent vaccine were protected from infection with *B. burgdorferi* N40. Antibody titers reached or surpassed the minimum previously shown to effectively protect against infection. Importantly, antibody titers were sustained for at least 6 months after immunization (Kamp et al., 2020), demonstrating the durability of a ferritin-based multivalent vaccine. This construct differs structurally from some Hpf constructs in that it is multivalent. The entirety of the OspA protein was thus used as opposed to an antigen epitope and demonstrates that large protein antigens can be stably conjugated to ferritin as well as small molecule agonists.

### Chitosan-based vaccines

Chitosan is a naturally occurring polysaccharide derivative of chitin, found in crustacean shells and fungi, which has many applications for vaccine delivery. Among these are a positive charge, mucoadhesive characteristics, and a highly basic nature. It is amenable to both chemical and physical modifications to enhance desirable vaccine properties (Mohammed et al., 2017). Chemical moieties can be conjugated at the amine and hydroxide groups, which makes it an excellent candidate for a vaccine carrier or adjuvant. When chitosan interacts with negatively charged mucus, a complex is formed which allows it to facilitate paracellular or transcellular transport across mucus membranes, thereby enhancing bioavailability. Chitosan NP (CNP) efficiently taken up by enterocytes and M cells in the gut and are also endocytosed by APC (Chen et al., 2013).

To further optimize uptake of CNP by APC, mannosylated chitosan (MCN) was utilized to take advantage of the high density of mannose receptors on macrophages and DCs. Binding to mannose receptors leads to endocytosis of the binding ligand and subsequently the subunit vaccine, which drives antigen presentation and T cell activation. In this study, authors utilized the FliC protein, a component of the flagellum, of *B. abortus* loaded on MCN, which was previously shown to contribute to survival of *B. abortus* and was identified as a potential vaccine candidate through *in silico* analysis (Vishnu et al., 2015). Mice given s.c. FliC-MCN demonstrated increased titers of IgG2a antibody compared with those that received peptide antigen only. This suggests a shift toward a Th1 biased response and potential for enhanced opsonization of bacteria. Similarly, IFN-γ and IL-2 production by splenocytes was higher in groups vaccinated with FliC-MCN compared with FliC, or MCN, alone. Interestingly, FliC-MCN did not significantly improve protection from *B. abortus* challenge compared with FliC alone as measured by splenic CFU of animals challenged with either *B. melitensis* 16 M or *B. abortus* 544 (Sadeghi et al., 2020). However, given that *B. abortus* is transmitted through mucus membranes *via* inhalation or ingestion, mucosal delivery of this vaccine may produce more favorable protective effects.

In a different approach, Shim, et al. utilized CNP conjugated to *B. abortus* malate dehydrogenase (Mdh) to evaluate activation of nasal-associated lymphoid tissue (NALT), and subsequent *B. abortus* Mdh-specific and total antibody production following i.n. vaccination (Shim et al., 2020). Transcriptomic analysis showed that the CNP-Mdh downregulated genes involved in cell viability, and upregulated inflammatory pathways, such as IL-6 signaling and HMGB1 signaling. Consistent with these pro-inflammatory signatures, genes associated with immune cell trafficking were also upregulated. The authors suggest that the CNPs were recognized by pathogen recognition receptors (PRR), which induce cytokine responses. Interestingly, the CNP-Mdh vaccination induced systemic mucosal immune responses as evidenced by the significant increase in Mdh-specific IgA antibodies 2 weeks post immunization in nasal washes, genital secretions, and fecal extracts compared with controls. Total IgG was significantly increased as well, which is evidenced by the parallel increase in IgG1 and moderate increase in IgG2a. These data indicate that the CNP conjugated to *B. abortus* Mdh triggered inflammatory signaling pathways, driving a mixed Th1/Th2 response and importantly, a robust mucosal antibody production (Shim et al., 2020).

One of the remarkable characteristics of chitosan is its amenability to various types of antigens, including DNA. DNA vaccines have been shown to effectively stimulate both humoral and cell-mediated immune responses, however, their limited stability results in low immunogenicity. Chitosan can be conjugated to DNA and provides protection from nuclease degradation. Feng, et al. demonstrated the use of chitosan to envelope and thereby protect a recombinant DNA plasmid containing three Mtb ESAT-6 T cell epitopes and further tested its immunogenicity and protective efficacy in an *in vivo* mouse model (Feng et al., 2013). Molecular studies of these constructs demonstrated that chitosan-enveloped DNA plasmids were not as easily degraded as those that lacked chitosan protection when exposed to DNAse I (Feng et al., 2013).

Stability provided by chitosan in a vivo model was further exhibited by an increase in ESAT6 peptide epitope expression in mice treated with a chitosan formulated vaccine compared with those that only received the DNA vaccine. Splenocytes from mice vaccinated with the chitosan-formulated DNA vaccine expressed superior levels of IFN-γ, IL-12, and T-bet mRNA when compared with s.c. BCG inoculation; however, BCG produced the highest IL-4 and IL-10. Importantly, the nanovaccine induced a significant reduction in Mtb CFU 4 weeks after intratracheal (i.t.) challenge (Feng et al., 2013). These results suggest a bias toward Th1 polarization in animals vaccinated with nanovaccines that induces protective responses in comparison to s.c. BCG. How a chitosan-based DNA vaccine might induce stronger mucosal responses if delivered through a mucosal route remains to be investigated and may be important for establishing tissue-resident immune and memory populations in common sites of infection, such as the lungs.

Further demonstrating the compatibility of chitosan with a broad array of antigens, CNPs have been used with Mtb lipids to generate specific γδ T cells and humoral immune responses, as demonstrated by Das et al. (2017). In this study, chitosan NPs were coated with Mtb lipids extracted from the cell wall and delivered s.c. over the course of several weeks. Mtb lipid-coated CNPs demonstrated significantly reduced cytotoxicity in macrophages compared with those treated with Mtb lipid liposomes. In mice, the lipid-coated CNPs elicited robust Th1, Th2, and pro-inflammatory cytokine responses from lymph nodes and spleen mononuclear cells when compared with PPD-treated controls. Enhanced antibody production, including total IgG, IgG1, and IgM, as well as a moderate increase in IgG2a and IgA, was also observed compared with controls (Das et al., 2017).

Shigellosis is an important global pathogen spread through contaminated food and water. *S. flexneri* targets the epithelia of the colon and are phagocytosed by APC. The bacteria escapes from the phagosome by an invasive protein dependent process and enters the cytoplasm. The type 3 secretion system (T3SS) consists of a needle-like structure with repeated MxiH units that assist in invasion of other host cells (Muthuramalingam et al., 2021). Following i.n. vaccination in mice, MxiH was shown to induce IgG production in serum (Gilavand et al., 2020). Titers were further increased in response to immunization with MxiH that was conjugated to chitosan NP (CS-MxiH; Gilavand et al., 2020). Importantly IgA titers were also increased in mice vaccinated with CS-MxiH, but not MxiH adjuvanted with Freund’s adjuvant, compared with controls that included animals vaccinated with mock (PBS) or bare CS. Serum levels of IFN-γ and IL-4 were also increased in mice vaccinated with CS-MxiH compared to those vaccinated with mock, bare CS, MxiH, and adjuvanted MxiH. Levels of IL-4 were similarly elevated in animals vaccinated with CS-MxiH or adjuvanted MxiH. The enhanced cytokine production and antibody titers indicated development of both cellular and humoral immunity. Challenge studies, however, revealed moderate and non-significant improvement in survival after vaccination with CS-MxiH compared with non-vaccinated controls (Gilavand et al., 2020).

CNPs are relatively easy to construct and can be conjugated to several types of antigens and even adjuvants. Although not all chitosan-based vaccines described were able to enhance protection against challenge in animal models of infection, the results demonstrate potential for generation of immune responses. When conjugated to immunogenic antigens, CNPs can delay antigen degradation, and promote diverse cell-mediated and humoral responses that could be further optimized to generate protection.

### Self-assembling peptide nanofibers

While many of the platforms discussed here are based on nanoparticles of natural or metal-based inorganic compounds, self-assembling peptides consist of non-native peptide sequences that form high aspect ratio nanofibers. A myriad of peptide antigens can be conjugated, to these self-associating peptides and the length, shape, and charge of the peptide backbone can be modified as well. Peptide nanofibers (PNF) are self-adjuvanting and capable of multivalency, which can be modified to include antigens in specific stoichiometric amounts (Rudra et al., 2012). The KFE8 PNF vaccine platform is composed of a short sequence of amino acids, FKFEFKFE, that forms β-sheet rich nanofibers and antigens conjugated to the N- or C-terminus of KFE8 are displayed on the nanofiber surface (O’Neill et al., 2021).

Our group has explored PNF-based vaccine platform as a booster vaccine following BCG inoculation to enhance and prolong immune responses against Mtb. We have shown that KFE8 fibrils are taken up by APC and processed through the autophagy pathway, driving antigen presentation (Rudra et al., 2017). Autophagy plays an important role in the presentation of cytoplasmic and nuclear antigens to the immune system. For example, it was previously shown to improve antigen presentation of mycobacterial antigens on MHC II molecules, which is a critical step in developing protective immune responses to Mtb (Crotzer and Blum, 2009). We previously demonstrated that KFE8 conjugated to immunodominant T cell epitopes from Mtb (TB10.4, ESAT6, or Ag85B, or multi-eptiope co-assemblies), generated Mtb antigen-specific CD4^+^ and CD8^+^ T cells that were capable of multifunctional cytokine production (Chesson et al., 2018). Specifically, when the TLR2 agonist Pam2CSK was incorporated into the PNF construct, APC enhanced expression of activation markers and inflammatory cytokines when compared with those treated only with agonist or with PNF. This further improved multifunctional T cell responses. Interestingly, when a CD4^+^ T cell epitope was used in combination with a CD8^+^ T cell epitope, overall CD8^+^ T cell responses were improved. In a challenge study, mice vaccinated with KFE8-Pam-TB10.4 following a BCG prime demonstrated a moderate decrease in Mtb CFU in the lungs at 4 weeks post infection in comparison with BCG alone (Chesson et al., 2018).

In a more recent study, we demonstrated that mucosal vaccination with KFE8-Ag85B in BCG-primed mice induced generation of heterogenous antigen-specific CD4^+^ T cells (Files et al., 2022). A subset of these cells was further characterized as a lung-resident memory T cell phenotype. These cells displayed surface molecules known to confer functional differences, such as CD49a and CD103, demonstrating the heterogeneity of the memory population. Although tissue-resident memory T cell (Trm) populations were primarily observed in the lung, antigen-specific splenic recall responses distinct from BCG suggests presence of vaccine-induced peripheral memory, as demonstrated by the increased production of IFN-γ and IL-18. Some mice received a DC vaccine pulsed with KFE8-Ag85B. This resulted in an enhancement of the effects seen in KFE8-Ag85B boosted animals, with the addition of increased Th1, Th2, and Th17 cytokines compared with BCG vaccination alone. Importantly, this study also demonstrated the effects of KFE8 nanofibers on the DC transcriptome, wherein we observed an upregulation of genes involved in responses to cellular stress, antigen presentation, detoxification, and mitochondrial respiration. Interestingly, we observed a lack of inflammatory cytokine upregulation. These results suggest that KFE8-treated DCs are responding to damage-associated molecular patterns (DAMPs), which may be a crucial component of the self-adjuvanting nature of KFE8 (Files et al., 2022). Taken together, this study demonstrated the importance of mucosal vaccination with KFE8 in inducing Trm populations in the lung. Other vaccine studies using the KFE8 platform have shown robust cell-mediated and humoral responses (Rudra et al., 2012; Friedrich et al., 2016; Rudra et al., 2018). Humoral responses to protein antigens are primarily T cell-dependent, thus, the ability of KFE8 to induce strong CD4^+^ T cell responses is critical to the development of observed humoral immunity (Rudra et al., 2012).

Other peptide nanofibers, such as Q11 (QQKFQFQFEQQ) have similar characteristics in that they are largely nonimmunogenic alone and immune responses are somewhat dependent on fibrilization of the peptide (Rudra et al., 2012). Q11 has yet to be utilized as a vaccine platform against intracellular bacteria; however, Q11 has been conjugated to a CD8^+^ T cell epitope derived from influenza polymerase. Q11-loaded DCs delivered i.n. were trafficked to lung-draining lymph nodes and generated significantly increased antigen-specific CD8^+^ T cell responses when compared with a Q11 construct delivered through a parenteral route. Importantly, these influenza-specific CD8^+^ T cells were shown to produce IFN-γ in response to antigen, have cytolytic activity, and decreased viral load in the lungs (Si et al., 2018). Additionally, the route of administration played an important role in generating antigen-specific lung-resident memory CD8^+^ T cells, while s.c. delivery generated greater numbers of antigen-specific circulating CD8^+^ T cells. In a similar study, i.n. delivery led to uptake of Q11 by APC, including CD103^+^ and CD11b^+^ DCs (Si et al., 2020), which have been shown in other studies to be important in generating protective immune responses against a variety of respiratory pathogens, including Mtb (Griffiths et al., 2016). Antigen presentation by Q11-primed DCs was shown to occur in draining lymph nodes and elicited a predominantly Th17 response. Taken together, the data show that pulmonary delivery of PNFs generates antigen-specific T cells responses in the lung and draining lung nodes. Coil29 is alpha-helical self-assembling peptide nanofiber that, although structurally different, demonstrated similar adjuvanting capacity and robust generation of cell-mediated and humoral responses (Wu et al., 2017). The inherent structural differences in these platforms may have differing effects on immune populations. While studies with both Q11 and KFE8 have demonstrated their applicability in vaccine design and importantly mucosal delivery (Si et al., 2018), it is imperative to understand the molecular characteristics underlying specific immune responses and how they can be leveraged to produce protective immunity.

In addition to cellular immunity, several designed PNF vaccines have been shown to induce highly protective antibody responses in preclinical models of infections and cancers (O’Neill et al., 2021). Malaria model experiments using the Q11 as the self-assembling motif elicited durable and protective anti-malaria antibodies. Fibrils composed of KFE8 linked to a cocaine hapten modified at the P3 site have been shown to boost anti-cocaine antibody titers without the need for exogenous adjuvants. Also, whole protein antigens delivered using depots of KFE8 or RADA peptide nanofibers admixed with viral coat proteins from West Nile virus or Hepatitis B virus resulted in significantly higher antibody titers in mice compared to the same proteins delivered using alum. As emerging evidence suggests that antibodies can protect against intracellular pathogens, such platforms may be beneficial to generate both humoral and cellular immunity to modify the course of intracellular bacterial infections.

### Charge-switching synthetic adjuvant nanoparticles

Mucosal vaccination has the advantage of triggering localized immune responses by targeting mucosal-associated lymphoid tissues. However, due to the differential permeability of mucosal surfaces, targeting APC, such as DCs at sites of infection at mucosal surfaces may be more difficult. Stary et al. demonstrated that incorporation of charge-switching synthetic adjuvant NPs (cSAPs) into a mucosal *C. trachomatis* (*Ct*) vaccine, led to development of a more effective immune responses to Ct challenge. cSAPs are formed from charge-switching synthetic particles (cSPs), which are composed of a hydrophobic core (PLGA and PLA) and a hydrophilic surface (PLH and PEG), although cSPs were originally formulated without the additional PLA (Radovic-Moreno et al., 2012). The additional PLA serves as a covalent linkage for the TLR7/8 agonist, R848. cSAPs carry a negative charge at physiological pH 7.4, and are conjugated to negatively charged bacterial surfaces following slight acidification to pH 6.5. Acidification of the cSPs results in the protonation of a poly(L-histidine) imidazole group, yielding a positively charged surface. The positively charged surface of the NPs allows greater interaction with mucosal surfaces. In this case, the cSAPs were conjugated to UV-treated *C. trachomatis* elementary bodies.

Intra-uterine vaccination with UV-Ct-cSAPs induced *Ct*-specific Th1 memory cells and resident memory T cells (Stary et al., 2015). These memory T cell populations have been shown to be required for clearance of Ct and generation of a robust antibody response, although the absence of B cells and CD8^+^ T cells does not have a significant impact on bacterial burden in animal models (Stary et al., 2015). Interestingly, UV-Ct-cSAP vaccine particles were preferentially taken up and transported to draining lymph nodes by CD103^−^ DCs expressing immunostimulatory markers, such as CD80 and CD86. The UV-Ct vaccine alone elicited a predominantly tolerogenic response and correlated with increased uptake by CD103^+^ DCs. This further resulted in upregulated immunoregulatory molecules associated with tolerance that corresponded to an increased burden of Ct and susceptibility to future infections (Stary et al., 2015). Together, these data provide an important proof of principle that mucosal vaccination with UV-Ct-CSAPs generates sufficient immune responses to overcome the tolerizing effect of killed Ct, which was previously a challenge to vaccine development. Future studies utilizing Ct-targeted cSAPs or other NPs in site-specific vaccination strategies should further characterize the mucosal immune responses that are generated.

### Polyhydroxyalkanoate biobeads

“Biobeads” are polyhydroxyalkanoate (PHA) granules that can be synthesized and functionalized inside genetically engineered bacteria with controlled diameters (0.1–100 μm), depending on the method of synthesis and functionalization. For example, Parlane, et al. found that PHA granules produced in *L. lactis* bacteria ranged from 50 to 150 nm in diameter, while granules produced in *E. coli* ranged from 150 to 250 nm (Parlane et al., 2012). The engineered bacteria are exposed to an excess of carbon and produce precursor molecules *via* encoded enzymes PhaA and PhaB, which are then polymerized by the enzyme PhaC, which remains covalently bonded to each polyester molecule that it polymerizes. These polyester-PhaC molecules self-assemble into beads with polyester cores and PhaC conserved on the surface (Parlane et al., 2014). By changing the genes encoding PhaC to produce a PhaC-antigen complex, antigen can be covalently bound to the surface of the Biobeads, effectively functionalizing them as particulate vaccines.

PHA NP vaccines targeting Mtb were engineered to display antigens Ag85A and ESAT6 and administered to mice s.c. Cytokine responses from splenocytes of vaccinated mice demonstrated development of antigen-specific recall including production of IFN-γ, IL-2, IL-6, TNF-ɑ, and IL-17A (Parlane et al., 2012). Biobeads have also been described to possess self-adjuvanting and immunomodulatory properties (González-Miró et al., 2018). In a *Neisseria menigitidis* model, mice vaccinated with Biobead-based NP displaying the NadA antigen, with or without an alum adjuvant, demonstrated that the inclusion of alum led to reduced serum IgG titers and cytokines. The is presumably due to loss of antigen display on the NP surface when emulsified with aluminum adjuvants. The NadA-functionalized beads induced Th1, Th2, and Th17 responses (González-Miró et al., 2018). In the context of a capsular polysaccharide antigen, a virulence factor of *N. menigitidis*, functionalized PhaC beads were found to induce more IL-17A and IFN-γ production than alum controls. These findings may indicate that PhaC beads possess intrinsic immunostimulatory properties and through mechanisms yet to be discovered, are capable of enhancing the immunogenicity of conjugated antigens.

In another vaccine study targeting Mtb, Chen, et al. utilized PHA NP as a carrier for Mtb immunodominant fusion proteins designated H4 (Ag85B and ESAT6) and H28 (Ag85B, ESAT6, and latency-associated antigen, Rv2660c; Chen et al., 2019). Importantly, mice vaccinated s.c. with H28-polyester NPs demonstrated significantly greater production of Th1 and Th17 cell cytokines (IFN-γ, TNF-α, IL-17A, and IL-2) in antigen recall assays 12 weeks after the last immunization in comparison with control mice. While there were no significant differences in comparison with those mice that received soluble H28; mice that received H28 particles (not conjugated to PHA) exhibited significant increases in IL-17A and TNF-α in comparison to the H28-polyester NPs. Following Mtb challenge, mice that received H4 or H28-polyester vaccines also demonstrated a significant reduction in lung CFU 6 weeks post-infection; although, there was no significant difference in comparison with BCG vaccination. In spleen, only mice that received BCG demonstrated a reduction in bacterial burden (Chen et al., 2021). Thus, PHA granules biosynthesized by genetically engineered bacteria hold promise as carriers for protein or peptide antigens to control intracellular bacterial infection. In the studies discussed here, Biobeads induced strong cell-mediated and humoral responses, however, their self-adjuvanting properties deserve further exploration. Performance might be improved by altering the route of vaccination, size of the PHA particle, or by combining with other adjuvants.

## Conclusion

A wide variety of NP technologies have been developed and are currently under investigation as vaccine platforms to target intracellular bacteria. These platforms can enhance penetrance of mucosal barriers, improve antigen presentation, and result in both cell-mediated and humoral immune responses known to be important mediators of protection. Because several of these bacterial diseases have respiratory manifestations, a concern with NP-based mucosal vaccines is the possibility of lung damage if delivered through a pulmonary route. To date, the NP-based platforms discussed here appear to be a safe method to deliver to mucosal surfaces and adjuvant vaccines. Current evidence demonstrates that PNFs do not promote inflammation in the lung following an i.n. or i.v. vaccination (Si et al., 2018; Marulanda et al., 2021). Histological examination of tissues from animals vaccinated with manganese iron oxide NPs revealed minimal inflammation in the lungs following i.n. vaccination and nonremarkable organ pathology showed signs of injury or trauma following s.c. vaccination. Microglia exposed to iron oxide NPs did not exhibit any reduction in viability (Wu et al., 2013), although, some increased hemolysis was observed at high concentrations of NPs (Neto et al., 2018). The basis for protection generated by nanomaterial-based vaccines remains to be fully characterized and is important for efforts to improve vaccine efficacy and define immune correlates with diagnostic value.

Several vaccines discussed here generate humoral and cellular immune responses that may serve as correlates or surrogates of protection and require further validation in animal models and human subjects. The functionality of vaccine-induced antibodies against intracellular bacteria, however, is poorly understood compared to extracellular bacteria and is an area that requires further investigation. Several of these NP platforms also induce robust cell-mediated immunity, including generation of classical Th1 and Th2 populations, Th17 cells, cytotoxic T cells, and lung resident T cells that display defined memory phenotypes. As with many vaccine platforms, the generation of these traditional humoral and cellular immune responses to NP-based vaccines do not always align with protection. An emerging area of research that could be harnessed to address this gap is that of non-traditional memory responses that are activated by natural infection or attenuated pathogens. An important example is the development of TII such as that described following vaccination with the BCG vaccine. The heterologous TII immune mechanisms that provide protection following epigenetic changes driven by BCG or other bacterial pathogens could potentially be targeted through NP vaccine platform design. In a very recent report, mice were shown to be protected from sepsis due to *E. coli* due to trained immunity generated by immunization with β-glucan coupled to an iron oxide NP (Pan et al., 2022).

The modular nature of NP construction where immunostimulatory components can be swapped with ease and the ability to engineer them with varying physical properties (charge, shape, size, degradability, hydrophobicity, etc.) provides flexibility to build vaccines that activate a myriad of host defense mechanisms including TII and classical acquired immunity. Additionally, the size and antigen content of each NP can be optimized to meet the specific needs of each vaccine. Some subunit vaccines have proven to be successful, however, protective responses to intracellular bacteria likely require a broad array of antigens. Future investigations and development of bacterial vaccines should take advantage of the amenability of several of these vaccine platforms to include a variety of peptide antigens, DNA, or lipids to induce robust immune responses currently only rivaled by live-attenuated vaccination.

## Author contributions

MF wrote the manuscript with assistance from JR, KK, and JE. The graphical abstract figure was designed by KK with content input from MF, JE, and JR. All authors contributed to the article and approved the submitted version.

## Funding

MF was supported by a predoctoral fellowship through the Sealy Institute for Vaccine Sciences at the University of Texas Medical Branch, Galveston, Texas. KK was supported by the Jack Kent Cooke Scholarship Program, and the Jack Kent Cooke Foundation Summer Internship Program, at Smith College, Northampton, Massachusetts. The research was supported by the Washington University McKelvey School of Engineering, Department of Biomedical Engineering Commitment Funds (12-360-94361J) and in part by the NIH/NIAID (R01 AI130278) to JR. We also acknowledge joint funding to JE and JR through NIH/NIAID (R21AI115302).

## Conflict of interest

The authors declare that the research was conducted in the absence of any commercial or financial relationships that could be construed as a potential conflict of interest.

## Publisher’s note

All claims expressed in this article are solely those of the authors and do not necessarily represent those of their affiliated organizations, or those of the publisher, the editors and the reviewers. Any product that may be evaluated in this article, or claim that may be made by its manufacturer, is not guaranteed or endorsed by the publisher.
